# The STEPWISE study: study protocol for a smartphone-based exercise solution for people with Parkinson’s Disease (randomized controlled trial)

**DOI:** 10.1186/s12883-023-03355-8

**Published:** 2023-09-12

**Authors:** Sabine Schootemeijer, Nienke M. de Vries, Eric A. Macklin, Kit C.B. Roes, Hilde Joosten, Larsson Omberg, Alberto Ascherio, Michael A. Schwarzschild, Bastiaan R. Bloem

**Affiliations:** 1 DisordersDonders Institute for Brain, Cognition and Behaviour, Department of Neurology, Center of Expertise for Parkinson & Movement Disorders, Radboud University Medical Center, Nijmegen, the Netherlands; 2 Harvard Medical School, Boston, MA USA; 3 Department of Neurology, Massachusetts General Hospital, Boston, MA USA; 4 Department of Health Evidence, Section Biostatistics, Radboud University Medical Center, PO Box 9101, Nijmegen, 6500 HB the Netherlands; 5 Department of Sports Medicine, Canisius Wilhelmina Hospital, Burgemeester Daleslaan 27, Nijmegen, 6532 CL the Netherlands; 6 Sage Bionetworks, Seattle, WA USA; 7 Harvard T.H. Chan School of Public Health, Boston, MA USA; 8 Mass General Institute for Neurodegenerative Disease, Massachusetts General Hospital, Boston, MA USA

**Keywords:** Physical activity, Exercise, Parkinson’s disease, Smartphone, Step count, Randomized controlled trial

## Abstract

**Background:**

Exercise has various health benefits for people with Parkinson’s disease (PD). However, implementing exercise into daily life and long-term adherence remain challenging. To increase a sustainable engagement with physical activity of people with PD, interventions that are motivating, accessible, and scalable are needed. We primarily aim to investigate whether a smartphone app (STEPWISE app) can increase physical activity (i.e., step count) in people with PD over one year. Our second aim is to investigate the potential effects of the intervention on physical fitness, and motor- and non-motor function. Our third aim is to explore whether there is a dose-response relationship between volume of physical activity and our secondary endpoints.

**Methods:**

STEPWISE is a double-blind, randomized controlled trial. We aim to include 452 Dutch people with PD who can walk independently (Hoehn & Yahr stages 1–3) and who do not take more than 7,000 steps per day prior to inclusion. Physical activity levels are measured as step counts on the participant’s own smartphone and scaled as percentage of each participant’s baseline. Participants are randomly assigned to an active control group with an increase of 5–20% (active controls) or any of the three intervention arms with increases of 25–100% (intermediate dose), 50–200% (large dose), or 100–400% (very large dose). The primary endpoint is change in step count as measured by the STEPWISE smartphone app from baseline to 52 weeks. For our primary aim, we will evaluate the between-group difference in average daily step count change from baseline to 52 weeks. For our second aim, measures of physical fitness, and motor- and non-motor function are included. For our third aim, we will associate 52-week changes in step count with 52-week changes in secondary outcomes.

**Discussion:**

This trial evaluates the potential of a smartphone-based intervention to increase activity levels in people with PD. We envision that motivational apps will increase adherence to physical activity recommendations and could permit conduct of remote clinical trials of exercise for people with PD or those at risk of PD.

**Trial registration:**

ClinicalTrials.gov; NCT04848077; 19/04/2021. Clinicaltrials.gov/ct2/show/NCT04848077.

## Introduction

### Background and rationale

The number of people with Parkinson’s disease (PD) has increased by 145% over the past 25 years [1], giving rise to what has been termed a “Parkinson Pandemic” [2]. People with PD experience both motor problems (such as bradykinesia, tremor or freezing of gait) and non-motor problems (such as constipation, apathy or cognitive impairment). There is some emerging evidence to suggest that exercise might slow the progression of motor symptoms [3, 4], possibly by facilitating neuroplasticity [5, 6]. Exercise may also improve various non-motor symptoms [7]. Finally, exercise and physical activity are associated with a reduced risk of developing PD [8], possibly via the same fact on neuroplasticity [5, 6]. While the positive effects of exercise are generally accepted, the volume and intensity of exercise that are needed to reach clinically significant effects remain unclear. Most research has focused on the effects of high-intensity aerobic exercise, which seems more effective than exercise at lower intensities [3]. On the other hand, evidence is emerging that the sheer volume of physical activities also matters [9], and simply increasing the volume of physical activities may be easier to reach and maintain for people with PD.

To establish the health benefits described above, it is necessary to engage regularly in physical activity over longer periods of time [10]. Long-term adherence is therefore required. To reach such a good level of adherence, interventions should be easily accessible (i.e., offered in the people’s own living environment), be adapted to personal preferences and capabilities, and be engaging (i.e., motivating and fun). Interventions that can be performed at a self-chosen time and place and that can be performed independently from healthcare professionals might reduce barriers to consistently engage in physical activity.

Recent innovations in digital technology, such as apps and sensors on smartwatches and smartphones, open up exciting avenues for remote interventions as well as remote monitoring of the outcome. Smartphone apps can improve people’s motivation to engage in physical activity by including goal tracking and rewards [11] and simultaneously measure the attained activity levels using the built-in sensors [12]. Approximately 86% of the world’s total population now has a smartphone [13], which also offers an unprecedented opportunity to reach even people living in remote areas and in developing countries who have limited access to healthcare or sports facilities.

So far, only one study has investigated the effectiveness of a (tablet-based) application in promoting physical activity in PD [14]. While this study showed that people with PD were satisfied with using an exercise app, no significant increase in physical activity was reported. Other studies in older adults showed that apps increased physical activity, but the interventions were of short duration (lasted for two to six months) [15]. So, even though innovative technologies are highly promising, changing physical activity behavior in the long term is still a major challenge and needs further study.

### Objectives

In this study, we investigate the feasibility of a smartphone app (Smartphone-Titrated Exercise in Parkinson’s With Incentive-Supported Engagement: STEPWISE app) to improve physical activity in people with PD. Our primary aim is to evaluate the between-group difference in average daily step count change from baseline to one year (52 weeks). Our secondary aim is to investigate the potential effects of the intervention on physical fitness and motor and non-motor function. Our third aim is to explore whether there is a dose-response relationship between volume of physical activity and our secondary endpoints.

### Trial design

**Fig. 1:**
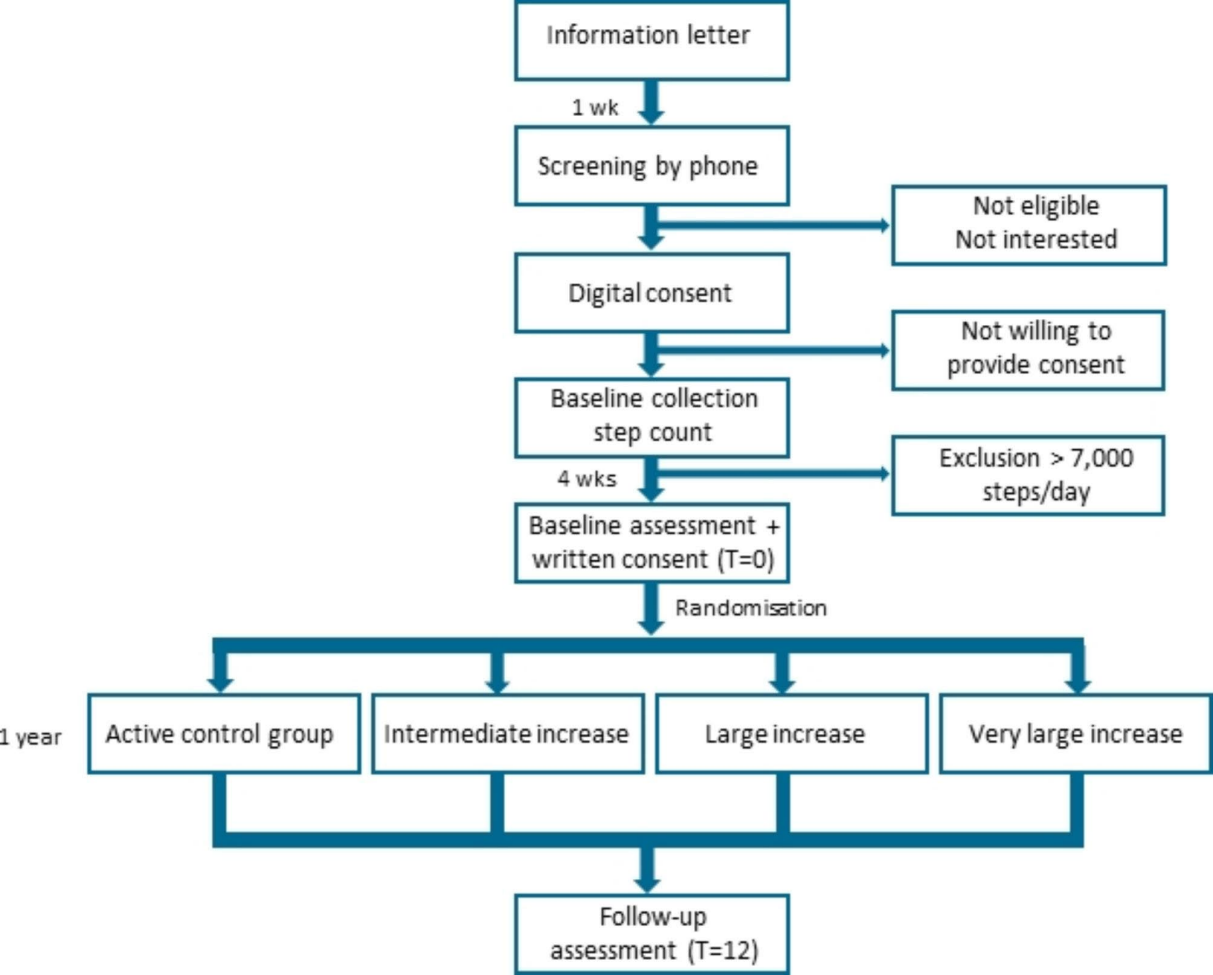
Participant flow chart

STEPWISE is a double-blind, randomized controlled trial in people with PD who perform a limited volume of physical activities. Participants are pre-screened and, if provisionally eligible, we determine their baseline step count over four weeks using the ‘STEPWISE app’ installed on the participant’s own smartphone. Participants have limited access to the app during this baseline period: they will only have access to their steps counted and will not be given any feedback except for their cumulative step count. If participants are determined eligible after this baseline period (see eligibility criteria), they complete a baseline set of assessments at Radboud University Medical Center (Radboudumc) and are randomized across four groups in a 1:1:1:1 ratio: the active control group (a small dose increase relative to their own step count at baseline), or to one of three intervention groups, each with a different dose increase (an intermediate, a large, or a very large dose increase relative to their own step count at baseline; Fig. 1). Participants return to Radboudumc for the assessment of secondary outcomes after 52 weeks.

The targeted daily step count will be determined by incrementing each participant’s baseline step count by a scaled proportion indexed by the participant’s random treatment assignment and time from baseline. The four groups correspond to the following percentage increase for a participant averaging 1000 steps per day at baseline (“base percentage increase”): 20% (active control group), 100% (intermediate group), 200% (large group), or 400% increase (very large dose group). To avoid excessively high target step counts, the target percentage increase is proportionally lower for participants with baseline step counts greater than 1000 (Eq. 1).

[1] Target percentage increase = base percentage increase * (baseline step count / 1000)^(-log_7_4).

This target percentage increase is approached linearly from baseline to the end of week 6 (Eq. 2). Beyond week 6, the daily step count target remains stable.

[2] Daily step count target = baseline step count * (1 + target percentage increase * ([increasing week number between 1 (week 1) and 6 (week 6 and beyond) / 6]))

**Fig. 2:**
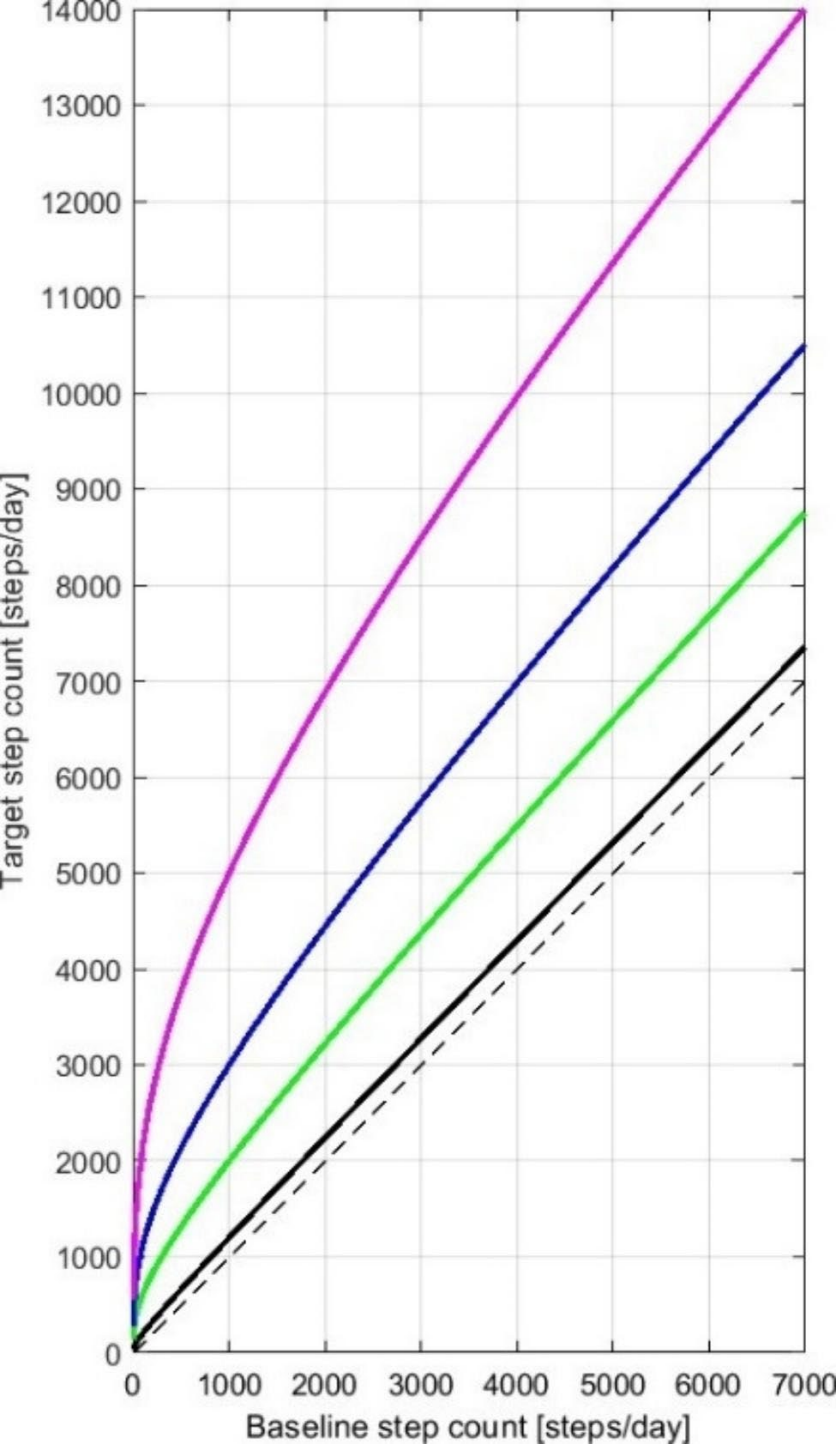
Target- and baseline step count. Dashed black line: line of identity. Black: active control group, green: intermediate dose, blue: large dose, purple: very large dose increase

For participants averaging 1000 to 7000 steps per day at baseline, the target daily step count for the active control group is a 5–20% increase, for the intermediate group a 25–100% increase, for the large group a 50–200% increase, and for the very large increase group a 100–400% increase (with smaller percentage increases for participants averaging more steps at baseline, Fig. 2). The 5–20% increase is considered an active control group given that a step count increase of this magnitude is expected not to be clinically meaningful.

## Methods: participants, interventions and outcomes

### Study setting

The study will be performed at Radboudumc and Canisius Wilhelmina Ziekenhuis (CWZ), Nijmegen, the Netherlands. Radboudumc is the study sponsor and is responsible for recruitment and inclusion of participants. Cardiorespiratory function will be assessed among a subset of participants at CWZ.

### Eligibility criteria

Inclusion criteria are: having a confirmed PD diagnosis according to the MDS criteria [16] by a neurologist, Hoehn and Yahr stage 1–3 during the clinical evaluation at baseline, being able to walk independently inside the home without the use of a walking aid, being able to understand the Dutch language, performing at most limited volume of physical activities prior to inclusion (i.e., taking fewer than 7,000 steps/day). People will be excluded if they have experienced weekly falls in the three months before enrollment, report medical conditions that hamper mobility other than PD, are not living independently, have cognitive impairments that hamper the use of a motivational app, or do not have a suitable smartphone (iPhone 5 S or newer with iOS (iPhone Operating System) 10 or higher or Android 4.1 or newer).

### Who will take the informed consent

As soon as possible upon registration, a description of the study is sent to potential participants by email, followed by a call one week later to discuss their interest and eligibility. During this phone call, potential participants are informed further about the study and the screening period, and are given the opportunity to ask questions. If they are provisionally eligible and willing to participate, informed consent is obtained in two steps (Fig. 1). First, participants sign a digital consent in which they agree to a four-week baseline/screening period. The step count eligibility criteria is checked during this baseline period. If they meet this criterion, they are invited to visit Radboudumc to be further assessed for inclusion. When eligible, a trained assessor will obtain written informed consent at the baseline visit before the start of the assessment. Participants sign the informed consent form in duplicate and take one copy home.

### Interventions

#### Intervention description

The intervention consists of a motivational app (STEPWISE app) that aims to motivate people with PD to walk more. We developed the STEPWISE app in close collaboration with people with PD. A pilot study was performed in which 30 people with PD used the STEPWISE app for four weeks. The qualitative feedback from this pilot study resulted in some minor revisions to the app. The development of the STEPWISE app and the results of the pilot study will be described in a separate publication (in progress).

**Fig. 3:**
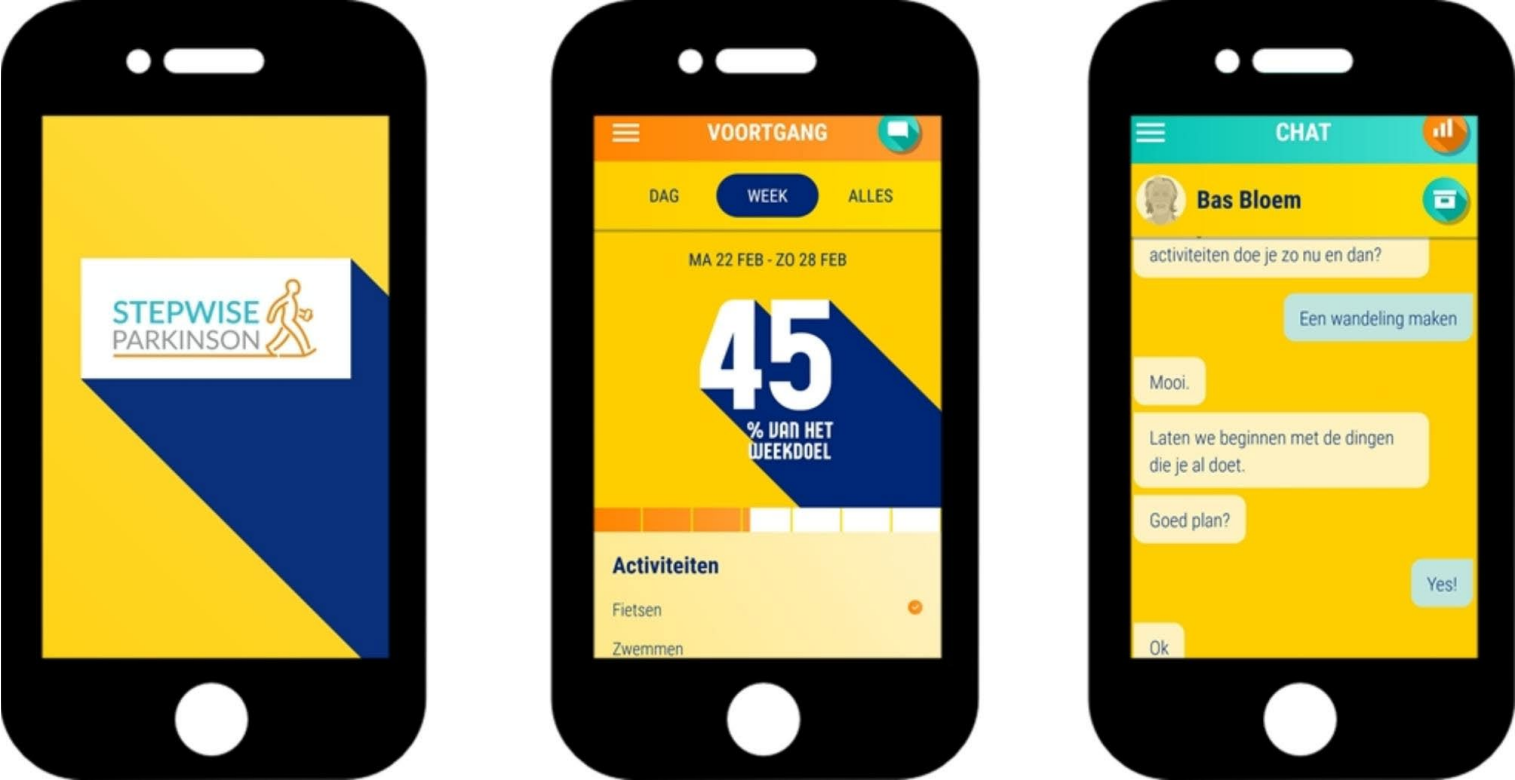
Screenshots STEPWISE Parkinson app. Splash screen, progression towards target, and chat with virtual coach

The STEPWISE app (Fig. 3) contains several motivational elements to increase engagement. First, participants are motivated by a virtual coach who gives support, provides tips to become (more) active, and shares knowledge about lifestyle and PD. Every other day, participants receive ‘text messages’ from this virtual coach to which they can reply with pre-set answer options. There is no real human interaction, but the different answer options result in different responses from the virtual coach. Importantly, the virtual coach gives participants the idea that they are being monitored by someone, which is a well-known motivator for people with PD [17]. Second, participants receive feedback on the achieved percentage of their weekly step count target. Their step count target is visualized as a percentage of steps taken towards their step count target every week. Participants also see the number of steps they took that day, the day before and the cumulative steps during the study. Participants are encouraged to reach 100% of their step count target every week. They see their progression as a percentage per week rather than as an absolute step count in order to blind them as much as possible. Moreover, they can decide themselves how they spread their physical activities over the week. Combining the visualization of the step count target and the virtual coach, we strived to create a balance between autonomy (where participants decide themselves when and how often they reach their target) and providing the idea of remote supervision (the virtual coach).

### Strategies to improve adherence to interventions

No strategies other than the STEPWISE app will be applied because we aim to evaluate the feasibility of a remote motivational app without human supervision or interaction. This is a pre-requisite for future use in larger populations and in more remote areas or in parts of the world with very limited access to healthcare facilities.

### Relevant concomitant care permitted or prohibited during the trial

There are no restrictions on usual care, but participants are asked not to participate in other interventional studies for the duration of their participation in the STEPWISE study.

### Outcomes

#### Primary outcome

The primary outcome is the mean within-participant change in step count per day from four weeks prior to the intervention (baseline period) to four weeks prior to the week 52 visit at the end of the intervention, as measured with the participants’ own smartphone. All randomized participants will contribute to the estimate of 52-week change in step count, including participants who terminate early, through the mixed model primary analysis (see below).

#### Secondary outcomes

The secondary outcomes that we will collect are listed in Table 1. During the in-clinic examination at baseline and after one year (52 weeks), we will collect: physical fitness (six minute walk test; 6MWT [18] and cardiopulmonary exercise test (CPET) in a subgroup of 100 participants), motor impairments (MDS-UPDRS-II and -III) [19, 20], mobility (Timed Up and Go) [18], balance (MiniBestTest) [18], gait speed (ten meter walk test (10MWT)) [18], handgrip strength (dynamometer), and cognition (Montreal Cognitive Assessment; MoCA)) [21]. In addition, the participants will receive the following online questionnaires that will be sent via our data management system (Castor EDC) [22]: fear of falling (Falls Efficacy Scale-I (FES-I)) [18, 23, 24], self-reported physical activity level (LASA Physical Activity Questionnaire; LAPAQ) [25], perceived physical activity (Lichamelijke Vaardigheden Schaal; LIVAS) [26], global non-motor symptoms (MDS-UPDRS-I) [20], motor complications (MDS-UPDRS-IV), depression and anxiety (Hamilton Anxiety and Depression Scale; HADS) [27, 28], apathy (Apathy Evaluation Scale; AES-12PD) [29], fatigue (Fatigue Severity Scale) [30], sleep (SCOPA-SLEEP) [31], autonomic dysfunction and constipation (Autonomic Questionnaire of the SCOPA; SCOPA-AUT) [32], health-related quality of life (Parkinson’s Disease Questionnaire; PDQ-39) [33], falls (monthly) and near-falls [34], barriers and motivators to engage in exercise (custom questionnaire), usability of the application (System Usability Scale) and global perceived effect (GPE) of the intervention. Participants who wish to complete the questionnaires on paper may do so.

As exploratory endpoints, we will assess PD motor symptoms remotely every three months using a second app (mPower) [35, 36]. In the mPower app, participants are asked to perform tasks that measure motor symptoms (i.e., tapping, walking, a tremor test) [35] and a cardiorespiratory fitness test (VO2max) [36] during quarterly bursts lasting 14 days. In addition, participants will wear an Axivity AX6 accelerometer for one week at baseline (between baseline visit and randomization) and for one week before the week 52 visit.

#### Participant timeline

Potential participants first enter a baseline period of four weeks in which we measure how many steps they take. As soon as possible (usually one to two weeks) after the baseline period, the first visit to the Radboudumc takes place where we perform a final eligibility check, collect secondary outcomes, and provide instructions about the study. All participants whose visit to the Radboudumc is scheduled at a timeslot that could include a cardiorespiratory function assessment are invited to enroll in the CPET sub-study at CWZ, up to a total of 100 participants. During the week after the baseline clinic visit, all participants wear a 6-axis inertial movement sensor at home to complete the baseline assessments. After all baseline assessments are complete, participants are randomized by a researcher not involved in assessments and given full access to the STEPWISE and mPower apps, which they will use for one year (i.e., 52 weeks; Fig. 1). Every three months, all participants who at the baseline visit provided consent to use the mPower app, receive a series of tasks offered by the mPower app during two weeks. One year after the baseline visit, participants will wear the 6-axis inertial movement sensor again for one week prior to their final follow-up visit at the Radboudumc. Participation and data collection is finished after this visit, unless participants wish to continue using the app. If so, a new informed consent is signed and data collection with the app is continued until the participant deletes the app or the complete study is finished. Participants who continue to use the app will be asked to complete the standard set of questionnaires (Table 1) every year.

**Table 1:** **Overview outcome measures.** Outcome measures with an asterisk will be collected with questionnaires which participants will fill in at home. Outcome measures with two asterisks are optional, if participants do not want to perform this assessment they can still participate in the study

Assessment	Baseline visit (T = 0)	During intervention	Follow-up visit (T = 12)
**Physical activity level**			
Step count with STEPWISE app (primary outcome)		x	
Physical activity with Axivity AX6	x (1 week)		x (1 week)
Self-reported physical activity level	x		x
**Physical fitness**			
6 minute walk test (6MWT) Cardiopulmonary exercise test (subgroup of 100 people) Remote cardiorespiratory fitness with mPower app **	x x x	x x x (1 day/3months)	x x x
**Motor symptoms**			
Timed Up and Go Test (TUG) Mini-BestTest MDS-UPDRS III (in on-state) MDS-UPDRS IV 10MWT (gait speed) Handgrip strength	x x x x x x		x x x x x x
Falls and near falls	x	X (monthly fall diary)	x
Falls Efficacy Scale - International (FES-I) * Lichamelijke Vaardigheden Schaal (LIVAS) *	x x		x x
Motor functioning with mPower app **	x	x (14days/3months)	x
**Non-motor symptoms**			
Montreal Cognitive Assessment (MoCA) Hamilton Anxiety and Depression Scale (HADS) * Abbreviated version of the Apathy Evaluation Scale (AES-12PD) * Scales for Outcomes in Parkinson’s Disease (SCOPA) * Fatigue Severity Scale (FSS) * MDS-UPDRS I and II *	x x x x x x		x x x x x x
**Quality of life**			
Parkinson’s Disease Questionnaire (PDQ-39) *	x		x
**Other**			
Custom questionnaire on blinding			x
Global Perceived Effect (GPE) * Custom questionnaire on barriers and motivators to engage in	x x		x x
physical activity			

#### Sample size

The planned sample size is 452 participants. This sample size is based on a previous study of one-year change in step counts in a clinical trial evaluating the use of a smartphone application to increase physical activity of patients with chronic obstructive pulmonary disease (COPD) [37]. Vorrink, Kort [37] reported a person-to-person standard deviation (SD) of change in one-year step count of 1957 in the active arm and 1973 in the control arm [38]. With 452 participants randomized 1:1:1:1 to the treatment arms (active control or intermediate, large or very large dose group), assuming an SD of 2000 steps and allowing for up to 20% loss to follow-up, the study will have greater than 90% power to infer a significant increase in step counts over one year if the expected 52-week increase in steps in the very large dose group relative to the active control group is at least 1000 steps based on a two-tailed test at p < 0.05 for this single primary comparison. One thousand steps is within the range of increases associated with exercise interventions among older adults and those with disabilities and chronic illness [39].

#### Recruitment

Participants will be recruited using multiple strategies. First, we will invite people with PD who are registered on the ParkinsonNEXT platform (N = 2,884; www.parkinsonnext.nl), which is an online platform that connects people with PD who are interested to participate in research with researchers and clinical studies. Second, we will advertise the study on social media (Facebook, Twitter, LinkedIn, Instagram) and on the website and newsletter of the Parkinson Vereniging (Dutch association for people with PD). We will also recruit through our outpatient clinic (neurologists and PD nurse specialists), via referrals from specialized physiotherapists who are part of the national ParkinsonNet (network of allied health professionals working with PD) [40] and by visiting Parkinson cafes (informative get-togethers for people with PD).

### Assignment of interventions: allocation

#### Sequence generation

Eligible participants will be randomized one week after their baseline visit. Eligible participants will be randomized in a 1:1:1:1 ratio to the small (active control), intermediate, large, or very large dose group using the Castor EDC data management system [21]. The randomization schedule will use random permuted blocks (block sizes: 4, 8, 12) stratified by sex (two groups: female and male) and disease duration (three groups: <5 years, 5–10 years, and > 10 years disease duration).

#### Implementation and concealment mechanism

Treatment assignment will be performed by Dr. Nienke de Vries (project leader), who is not involved in the intervention or data collection. Group allocation is concealed for all other members of the study team.

### Assignment of interventions: blinding

The study is double-blind meaning that the participants and the researchers are blinded to group allocation. The randomization is entered in the back-end of the app, whereafter participants have full access to the app. The app looks similar for participants in all groups to ensure blinding. Participants are unaware of the details of the allocation options: we tell participants that they will be randomized to one of four groups that are all motivated to take more steps, but to a different degree. Blinding of participants will be checked at the follow-up assessment at one year by asking participants whether they think were randomized to a group with a small or a large increase in step count. The blinding will not be broken prior to data lock unless the accredited medical research ethics committee (MREC) requests this.

### Data collection and management

#### Plans for assessment and collection of outcomes

The primary outcome (within-participant change in step count from baseline to the week 52 visit) is collected continuously over one year with participants’ own smartphones. Secondary outcomes are collected by trained staff in the Radboudumc or through questionnaires that participants complete at home via our data management system Castor EDC [22]. The questionnaires are sent to participants’ email addresses directly after each visit. If a participant wishes to complete the questionnaires on paper, they may do so. The cardiorespiratory fitness test is performed by trained staff at CWZ and results are entered directly into Castor EDC [22].

#### Plans to promote participant retention and complete follow-up

To promote retention, we make an appointment for the follow-up visit at baseline and call participants one month before the follow-up visit to remind them. Updates on the study are sent through the STEPWISE app.

#### Data management

The step count data in the STEPWISE app are collected locally (on the smartphone itself) through the HealthKit (iPhone) and Google Fit platform (Android). For Android users, the step count data are coupled to participants’ Google-account. We do not save any information other than the step count data. External parties possibly also have access to the physical activity data that is coupled to participants’ Google-account. The participants are informed about this in the study information and on the informed consent form. The step count data are stored with a unique personal identification code on a server hosted by Rootnet B.V. The server is protected by software updates, daily backups and 24/7 monitoring conforming to ISO27001 and NEN 7510.

Secondary outcome measures collected in the Radboudumc will be directly entered in the secured and certified data management system Castor EDC [22] during the visit. Castor EDC also provides an audit trail. Data collected through the mPower app will be accessible for the researchers via Sage Bionetworks Synapse analysis platform. More information on data management and security of the mPower app is provided in the protocol of the “mPower Progression Study” (dossier number: 2021–8104).

#### Confidentiality

Personal information will be kept in our participant registration system (Salesforce, San Francisco, CA, USA). The participant registration system is password protected and only accessible to the research team. Before we collect participants’ step count for four weeks during the eligibility check, potential participants will get a personal unique identifier consisting of three random letters and numbers (e.g. AZE910). The coded data (personal information and their unique identifiers) will be kept separately from the experimental data. The experimental data will be kept in Castor EDC [22], which is username and password protected. The data will be locked and stored for 15 years (digitally or on paper) after the study.

### Statistical methods

#### Statistical methods for primary and secondary outcomes

Data will be analyzed according to the intention-to-treat principle.

#### Primary outcomes

The primary endpoint is within-participant change in daily step counts. We will evaluate the between-group change in step counts, comparing the active control group and each interventional group (intermediate, large, very large dose). The mean daily step count in the four-week baseline period will be compared to the mean daily step count in the four weeks prior to the week 52 visit (weeks 49–52). We will analyze participants’ mean daily step count during each 4-week interval starting with the 4 weeks prior to baseline and ending with the 4 weeks prior to the week 52 visit in a shared-baseline, mixed model repeated-measures (MMRM) analysis. The model will include fixed terms of observation interval (14 terms), treatment group x target step count x post-baseline interval interaction (39 terms), age x pre/post-treatment interaction (2 terms), and disease duration x pre/post-treatment interaction (2 terms). Covariance among the within-participant repeated measures will be assumed to be unstructured. The primary estimate will be the one degree of freedom linear contrast tested at two-tailed p < 0.05 comparing change from baseline to the final four weeks (week 49–52) between the group randomized to a very large dose increment in steps vs. the active control group. Secondary assessments will compare the intermediate and large dose increment groups to (a) the active control group to determine whether smaller increments also result in measurable increases in physical activity and (b) the very large dose increment group to determine whether a ceiling effect is reached. Additional analyses will consider group-dependent changes in step counts from baseline to 52 weeks of follow-up to identify temporal patterns of response to determine whether larger increments occur early that are not sustained to the final four weeks of the intervention. Additional sensitivity analyses will consider alternative covariance structures, e.g., compound symmetry, implying a single random participant-specific intercept. Exploratory analysis will use time-dependent mediation analysis to identify the specific game elements that were associated with the largest maximal and longest sustained increases in step counts.

#### Secondary outcomes

The secondary study parameters are within-participant change in physical fitness, motor impairments, non- motor symptoms, and health-related quality of life. We will use equivalent analyses as we used for the primary analysis but with fewer observation intervals to estimate treatment-associated differences in one-year change in these secondary outcomes. Significant improvement when assigned to an intermediate increment or greater (all three interventional arms combined) at one-tailed p < 0.20 will be taken as evidence of preliminary effectiveness.

In exploratory analyses, we will relate the change in step count from baseline to 52 weeks follow-up to the 52-week change in clinical outcomes. To test whether there is a dose-response relationship between amount of physical activity and physical fitness and motor- and non-motor functioning, we will regress 52-week change in clinical outcomes against 52-week cumulative step count, adjusting for age, disease duration, baseline VO2max, and baseline step count. We will evaluate the clinical relevance of the association of the step count with clinical outcomes through estimated effect sizes based on the regression terms, including 95% confidence intervals. In secondary analyses, we will determine the threshold of physical activity leading to clinically relevant changes using a generalized additive model with each of the predictors above modeled as low degree of freedom monotonic splines. The 52-week increment in step count yielding a minimum clinically important increment will be interpolated from the spline for 52-week change in step count, with a confidence interval estimated by bootstrapping.

#### Other study parameters

Baseline characteristics will be summarized as counts and percentages or as means, medians, standard deviations, and ranges.

#### Interim analyses

One interim analysis will be performed to ensure that the app leads to a sustained increase in step count in the interventional groups (intermediate, large and very large dose) and at most a modest increase in step count in the active control group. We will not terminate this study based on the results of these analysis. However, we may change the intervention (the app) or the design of the study if the intended titration of increments deviates more than set thresholds given below. The interim analysis will be performed when the dataset consists of (at least) 100 participants with 3 months of follow-up. This would be at roughly 600 participant-months of follow-up. We defined the following thresholds for potential revision of the intervention / app:

1) If fewer than 40% of participants in the intervention arms increase their step count by more than 20%, we need to revise the app;

2) If more than 40% of participants in the control arm increase their step count by more than 35%, we need to revise the app.

There may be other (technical or operational) reasons to adjust the app. If changes are made to the app following the interim analysis, then primary inference will be made from data collected after any change is implemented. The interim analysis requires partial unblinding (control versus intervention groups). To safeguard blinding of all researchers directly involved in the trial, the interim analysis will be performed by staff not directly involved in operations.

#### Plans to give access to the full protocol, participant level-data, and statistical code

Meta data will be shared in an online repository. The full protocol is posted at ClinicalTrials.gov (NCT04848077).

### Oversight and monitoring

#### Composition of the coordinating centre

Data quality and safety will be monitored by an independent monitor from the Radboudumc according to the guidelines of the Nederlandse Federatie van Universitair medische centra (NFU).

#### Adverse event reporting and harms

Adverse events are defined as any undesirable experience occurring to a participant during the study, whether or not related to the use of the motivational application. Adverse events for which the participant obtains a medical check-up and are reported spontaneously by the participant and adverse events observed by the investigator during in-clinic visits will be recorded. We will ask participants to report falls using a monthly fall diary sent through Castor EDC [22]. Falls are considered an adverse event. A serious adverse event (SAE) is any untoward medical occurrence that results in death, is life threatening, results in hospitalization or prolongs an existing hospitalization, or results in persistent/significant disability or incapacity. Medical events that did not result in any of the outcomes listed above due to medical or surgical intervention but could have had these outcomes based on judgment of the investigator are SAEs. An elective hospital admission is not an SAE.

We will report all SAEs through the web portal ToetsingOnline to the accredited medical ethics committee that approved the protocol. We will do so within 7 days of our first knowledge of SAEs that result in death or are life threatening. After reporting, we will submit a preliminary report within 8 days. All other SAEs will be reported within 15 days of our first knowledge of the SAE.

#### Plans for communicating important protocol amendments to relevant parties (e.g.,. Trial participants, ethical committees)

Substantial amendments to the research protocol are only implemented after approval by the medical ethics committee. Participants will be informed when substantial amendments are made to the participant information or the informed consent. Non-substantial amendments will not be submitted to the medical ethics committee or competent authority, but will be recorded and filed by the sponsor.

#### Dissemination plans

The results of this study will be communicated to people with PD, professionals working with PD, and to the general public. People with PD, including participants in this study, will be informed about the results of this study through email, a web-based television program (www.ParkinsonTV.nl/www.parkinsonTV.org), publications on an online community for people with PD, their partners, and healthcare professionals (www.ParkinsonConnect.nl), newsletters and magazines of national and international PD patient associations. Professionals working with PD, the broader scientific community, health care professionals, and the general public will be informed about the results of the study through ParkinsonNet, PD patient associations, publications in the scientific literature, presentations at national and international conferences, and via social media.

## Discussion

The STEPWISE study will collect a unique dataset comprising continuous step count data of a planned group of 452 participants over one year to study the feasibility of a motivational smartphone app to increase the physical activity level of people with PD. We will test the effects of assignment to increased physical activity, as supported by the smartphone app, the change in PD motor- and non-motor symptoms after one year. We will also investigate any dose-response relationship between the achieved increase in volume of activity and subsequent changes in fitness as well as motor and non-motor function. We expect that this trial will help to determine whether and how to scale the STEPWISE study to a global, (fully) remote trial of physical activity for PD.

Our study has several strengths. First, one of the unique elements of the STEPWISE study is that it is almost completely performed remotely. While this has been done before in other conditions, and with pharmacological trials [41], this is an innovative approach in the PD field and for non-pharmacological interventions. While participants visit the clinic only twice to collect secondary outcomes, the intervention is delivered completely remotely without human interaction, in the comfort of participants’ own home and using their own smartphone. This same smartphone is also used to collect the primary outcome (step counts) and a range of secondary outcomes (collected through the mPower app). Second, we measure participants’ physical activity level objectively, continuously, and for one year, which will give us insight into actual physical activity behavior in the home environment during a prolonged period. Apps targeting physical activity (albeit in the general population) have proven effective in the short term (e.g., over a maximum period of 3 months) [38], but long-term effects have not yet been shown. We intend to fill this gap in order to establish the feasibility and methodology of generating long-lasting, meaningful effects. In addition, previous studies have mainly measured physical activity at specified time points (e.g., one week at the start and after the end of the study) [14], which could influence the results since participants know that they are being “watched” by the investigators during these times, or with questionnaires [42], which are prone to recall bias. Third, we will measure physical activity with the participants’ own smartphone and not an additional device, such as a smartwatch or dedicated fitness device, which has the advantage that participants only have to use one device (and importantly, their own device to which they are fully accustomed). Also, not everyone can afford or wants to have a smartwatch, so reliance only on smartphones may reduce inclusion bias. We envision this approach to be more scalable, opening up avenues for future research in more remote areas. Fourth, we will investigate a possible dose-response relationship between volume of physical activity and secondary outcomes such as PD motor- and non-motor features which has not been well studied in PD [43].

There are also a few potential challenges that we anticipate. First, recruitment of 452 participants who are motivated to enroll in an exercise study but who do not meet our exclusion criterion of a baseline step count greater than 7,000 steps per day might be challenging. Our center has an excellent track record of including participants in large clinical studies and -trials [4, 44–47], so we expect to be able to succeed in including such a large population of participants. Second, selection bias is a potential concern since participants who sign-up for the study will probably have an interest in physical activity or mobile technology. We strive for an inclusive and representative sample that is well-balanced between males and females and that includes people with PD who are less physically active, have limited experience with apps, have differing educational levels, and are of different ethnicities. To reach this aim, we will adopt recruitment strategies that directly target people with PD through both direct contact, selective advertising, and engagement of their clinicians. Third, the selection of the subgroup of 100 participants who perform a cardiorespiratory function assessment is pragmatic. We will monitor whether the sample enrolled in this CPET sub-study at CWZ is representative (in terms of sex, age, and disease duration). Fourth, a drawback of using step count as the main outcome is that step counts do not offer a measure of exercise intensity, and therefore we cannot include exercise intensity in our dose-response analysis. The literature indicates that exercise intensity is one of the factors that determines the efficacy of physical activity [3], although observational studies also indicate that simply a larger volume of physical activities is associated with reduced risk for PD [8, 9] and with milder disease progression [48]. Motivated by the latter findings, we will test whether attaining a higher volume of physical activity confers clinical benefits to our participants. Fifth, our intervention is limited to step-based physical activities. Participants are encouraged to perform any type of physical activity, but feedback is provided only with respect to their step count target. We appreciate that participants have different exercise preferences, which cannot all be measured with the app (e.g., swimming, biking). To partially address this concern, participants can enter any additional activities using free text in the app, but these will not be stored nor analyzed in this study. Lastly, we will measure step counts with the participants’ own smartphone. We appreciate that this may lead to an underestimation of actual activity because people sometimes forget to bring their phone [49] and that smartphones may differ with respect to the validity and reliability of determining step counts [50]. Importantly, however, our primary endpoint is within-participant (i.e., relative) change in step count. Even though estimates of absolute step counts may vary depending on the specific device being used, we do not expect that any imprecision will change over the course of one year, so the within-person change in step count may still provide an accurate estimate of their change in physical activity. We expect that the wide range of intervention targets will allow us to identify differences in outcomes across the groups even with some imprecision in absolute step counts.

In conclusion, this study will test the potential of a smartphone app to increase physical activity levels in approximately 452 people with PD for one year. We will determine the efficacy of different dose increases on one year change in PD motor- and non-motor symptoms and investigate the dose-response relationship between volume of activity and change in fitness and function. We envision that the STEPWISE study will inform many future trials on whether and how to scale the STEPWISE approach for future remote trials of physical activity for people with a chronic disease and potentially as an intervention that might slow disease progression in people with either prodromal parkinsonism or manifest PD.

### Trial status

The first participant was included on July 19, 2021. As of May 8 2023, 239 participants have been randomized of which 75 finished the study. We expect to randomize the last participant by the end of 2024. The current protocol version is version 10 (October 5, 2022).

## Data Availability

We will make the aggregated data available to other researchers upon request.

## Abbreviations

AES-12PD: Apathy Evaluation Scale
COPD: Chronic Obstructive Pulmonary Disease
CPET: Cardiopulmonary exercise test
CWZ: Canisius Wilhelmina Ziekenhuis
FES-I: Falls Efficacy Scale-I
GPE: Global Perceived Effect
HADS: Hamilton Anxiety and Depression Scale
iOS: iPhone Operating System
LAPAQ: LASA Physical Activity Questionnaire
LIVAS: Lichamelijke Vaardigheden Schaal
MoCA: Montreal Cognitive Assessment
MREC: Medical Research Ethics Committee
NFU: Nederlandse Federatie van Universitair medische centra
PD: Parkinson’s disease
PDQ-39: Parkinson’s Disease Questionnaire-39
SAE: Serious Adverse Event
SD: Standard Deviation
6MWT: Six minute walk test
10MWT: Ten meter walk test
